# Experimental Investigation of the Magnetoelectric Effect in NdFeB-Driven A-Line Shape Terfenol-D/PZT-5A Structures

**DOI:** 10.3390/ma12071055

**Published:** 2019-03-30

**Authors:** Juanjuan Zhang, Yan Kang, Yuanwen Gao, George J. Weng

**Affiliations:** 1 Key Laboratory of Mechanics on Environment and Disaster in Western China, The Ministry of Education of China, Lanzhou University, Lanzhou 730000, China; zhangjuanjuan@lzu.edu.cn (J.Z.); ywgao@lzu.edu.cn (Y.G.); 2 Department of Mechanics and Engineering Science, College of Civil Engineering and Mechanics, Lanzhou University, Lanzhou 730000, China; ykang13@lzu.edu.cn; 3 Department of Mechanical and Aerospace Engineering, Rutgers University, New Brunswick, NJ 08903, USA

**Keywords:** magnetoelectric effect, multiple resonance peaks, broadband response

## Abstract

In this paper, the magnetoelectric (ME) effect is investigated in two kinds of A-line shape Terfenol-D/PZT-5A structures by changing the position of the NdFeB permanent magnet. The experimental results show that both ME composite structures had multiple resonance peaks. For the ME structure with acrylonitrile-butadiene-styrene (ABS) trestles, the resonance peak was different for different places of the NdFeB permanent magnet. Besides, the maximum of the ME coefficient was 4.142 V/A at 32.2 kHz when the NdFeB permanent magnet was on top of the Terfenol-D layer. Compared with the ME coefficient with a DC magnetic field, the ME coefficient with NdFeB magnets still maintained high values in the frequency domain of 65~87 kHz in the ME structure with mica trestles. Through Fourier transform analysis of the transient signal, it is found that the phenomenon of multiple frequencies appeared at low field frequency but not at high field frequency. Moreover, the output ME voltages under different AC magnetic fields are shown. Changing the amplitude of AC magnetic field, the magnitude of the output voltage changed, but the resonant frequency did not change. Finally, a finite element analysis was performed to evaluate the resonant frequency and the magnetic flux distribution characteristics of the ME structure. The simulation results show that the magnetic field distribution on the surface of Terfenol-D is non-uniform due to the uneven distribution of the magnetic field around NdFeB. The resonant frequencies of ME structures can be changed by changing the location of the external permanent magnet. This study may provide a useful basis for the improvement of the ME coefficient and for the optimal design of ME devices.

## 1. Introduction

Magnetoelectric (ME) effect is defined as a magnetization response to an applied electric field or an electric polarization response to an applied magnetic field [1,2,3,4]. It exists in single materials and ME composites composed with ferroelectric and ferromagnetic materials [5,6,7,8]. Due to the higher ME coefficient in ME composites at room temperature, the ME composites have potential applications in many smart devices, such as magnetic field sensors, current sensors, energy harvesters, memory devices, filters, etc. [9,10,11,12,13,14,15,16]. In recent years, it has become a research hotspot in mechanics, physics, and materials science [17,18,19,20,21].

For the conventional laminated ME structures, the ME coupling characteristics can be affected by many factors, such as material performance, magnetic field, temperature field, and interface coupling performance [22,23,24,25,26,27,28,29,30,31,32,33]. Because the elongation of the ferroelectric layer is not directly produced by the applied electric field but instead driven by the ferromagnetic layer, the interface coupling between the two phases becomes the key factor for an enhanced ME effect [34]. In order to reduce the influence of interface properties on the magnetoelectric response, many novel magnetoelectric structures are designed and prepared [34,35,36,37,38,39]. For example, Bi et al. made the magnetic material into a groove and placed the piezoelectric layer in the groove. The magnetostrictive strain is transmitted to the piezoelectric layer through the boundary. This design makes the stress transfer between the magnetostrictive layer and the piezoelectric layer no longer dependent on the interface layer, avoiding the influence of the interface layer on the magnetoelectric response [36]. Zeng et al. reported a giant magnetoelectric effect in negative magnetostrictive/piezoelectric/positive magnetostrictive semiring structures, with the maximal ME coefficient at 56.3 V/A [38]. Wen et al. presented a novel ME structure, made up of one Terfenol-D layer and three PZT-5A layers, and showed that the three PZT-5A layers can be driven by one Terfenol-D layer simultaneously. The experimental results showed that multiple resonant frequencies and broad bandwidth characteristics existed in this ME structure [39].

All of the above experimental results require the application of a bias magnetic field to get a higher ME coefficient because the DC magnetic field has a significant impact on output strains of magnetostrictive materials [18,23]. However, the applied bias magnetic field is typically provided by electromagnets [34,40]. Usually, the electromagnetic device is very large, which not only increases the size of the structure but also the cost of the structure. Besides, due to the presence of an electromagnetic device, additional electromagnetic fields add a potential noise source [41].

In order to eliminate the large volume and electromagnetic interference of electromagnets and meet the demand for DC magnetic field in magnetoelectric experiments, permanent magnets have become a good substitute. There are many permanent magnets in nature. NdFeB is one of them. NdFeB magnets have the characteristics of being small in size and light-weight and having strong magnetic properties. They are widely used in magnetic experiments [42,43]. In ME experiments with NdFeB as a magnetic material, its addition will not only change the composition of the entire magnetoelectric structure; it will also have a great influence on the magnetic field distribution of the structure. Many researchers have done research in this area [44,45,46,47,48,49]. For example, Zeng et al. reported a unique ME effect in a device of PZT drum transducer and Fe-core solenoid [45]. Liu et al. have proposed a PZT ceramic fibers/phosphor copper-sheet composite beam with NdFeB magnets. This ME composite structure possesses colossal low-frequency ME coupling and brings about a remarkable enhancement in ME coefficients in the low frequency range [46,47]. Han et al. have proposed a nonintrusive method to realize self-powered sensors based on a magnetoelectric energy harvester that consists of a piezoelectric bimorph and NdFeB permanent magnets [48,49].

In this study, the ME effect was investigated in an A-line shape ME structure which was made up of a PZT-5A layer, Terfenol-D layer, and the trestles without magnetic and electric characteristics. Two kinds of ME structures were prepared by choosing ABS and mica materials as the trestles, respectively. A NdFeB permanent magnet with a small size was introduced in the A-line shape ME structure. The frequency dependences of the ME coefficient for the ME structures were studied by first changing the position of NdFeB permanent magnet. Then the structural frequency response characteristics were analyzed at different applied frequencies. Finally, a finite element analysis was performed to evaluate the resonant frequency and to analyze the magnetic field distribution in the A-line shape structures.

## 2. Experiment Details

### 2.1. ME Structures and Working Principle

**Figure 1:**
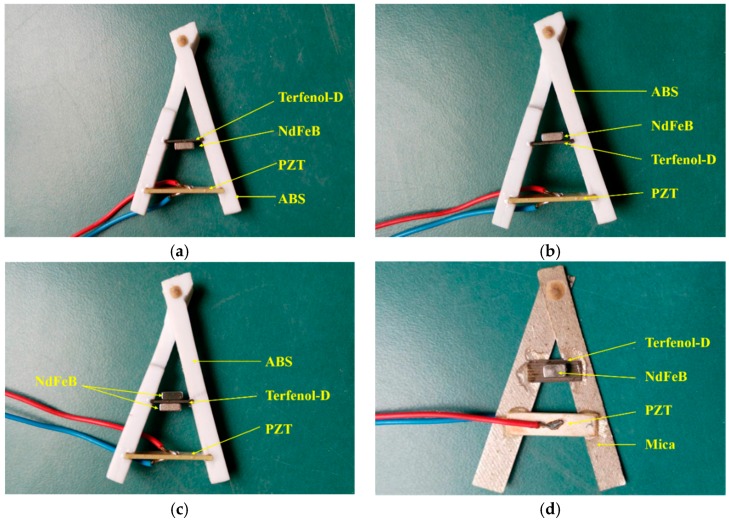
Photograph of the A-line shape Terfenol-D/PZT-5A structure with NdFeB magnets (**a**) ABS- case; (**b**) ABS+ case; (**c**) ABS+- case; (**d**) Mica case.

**Figure 2:**
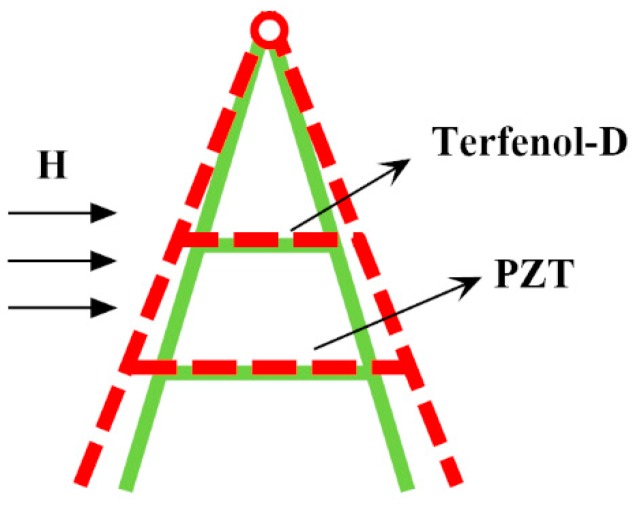
The working principle of A-line shape ME structures.

**Figure 3:**
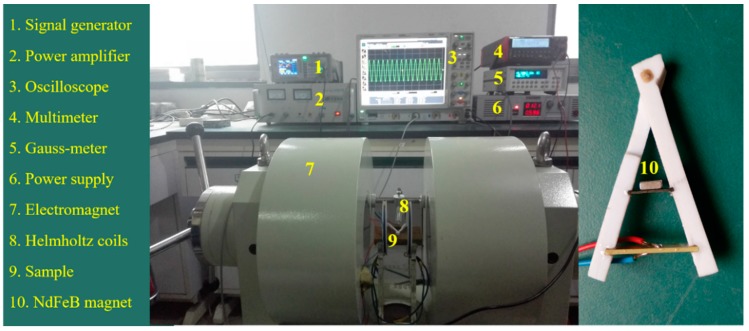
Physical drawing of ME test system.

In our experiment, the ME structures were made of piezoelectric material, magnetostrictive material, permanent magnet, and the trestles which were without any magnetic and electric characteristics. A PZT-5A layer (Shouguang Feitian Electronic Co. Ltd., Shouguang, China) and Terfenol-D layer (Tianxing Company, Lanzhou, China) were selected as the piezoelectric and magnetostrictive materials. The dimensions of the two materials were 20 × 5 × 1 mm^3^ and 12 × 5 × 0.5 mm^3^, respectively. The polarization was perpendicular to the PZT layer (i.e., thickness direction), and the magnetization was along the longitudinal direction of the Terfenol-D layer. Then we chose ABS resin and mica flakes as the trestles and prepared two types of ME structures. The trestles were hinged at the top. For the ME structure with ABS trestles, the end of PZT-5A and the Terfenol-D layers were embedded in the ABS trestles, the PZT-5A layer, and the Terfenol-D layer, which were parallel. The NdFeB permanent magnet (Jiuci Magnetic Materials Co., Ltd., Beijing, China) was placed in different locations of the Terfenol-D layer. The size was 5 × 3 × 2 mm^3^. We measured three different situations, as shown in Figure 1a–c: (1) the NdFeB permanent magnet was placed under the Terfenol-D layer (see Figure 1a); (2) the NdFeB permanent magnet was placed on top of the Terfenol-D layer (see Figure 1b); (3) the NdFeB permanent magnet were placed on the top of and beneath the Terfenol-D layer (see Figure 1c). For convenience, we denote the three conditions above as ABS- case, ABS+ case, and ABS+- case. For the ME structure with mica trestles, the edges of the PZT-5A and Terfenol-D layers were separately bonded with the mica trestles, and the NdFeB permanent magnet was placed on top of Terfenol-D layer, as shown in Figure 1d. The PZT-5A layer and Terfenol-D layer line were on the same plane and parallel to each other.

The working principle of A-line shape ME structures is shown in Figure 2. When the ME structure was in an external magnetic field, the Terfenol-D layer elongated along the length direction due to the magnetostrictive effect. Because the ends of the Terfeonl-D and PZT layers were fixed on the trestle, the large PZT layer could be driven by the small Terfenol-D layer, and it also elongated. The deformation of the PZT layer caused a polarization electric field because of the piezoelectric effect. The ME coupling process of the whole ME structure was completed.

### 2.2. Measurement Setup

In general, the applied magnetic field included DC and AC parts in the ME experiment. The DC magnetic field was usually provided by an electromagnet and a power supply, see Figure 3. Their volumes were large, which increased the size of the ME devices and added a potential noise source. In our experimental tests, instead of an electromagnet and a power supply, the DC magnetic field was generated by the NdFeB magnet with a smaller size (5 × 3 × 2 mm^3^).

**Figure 4:**
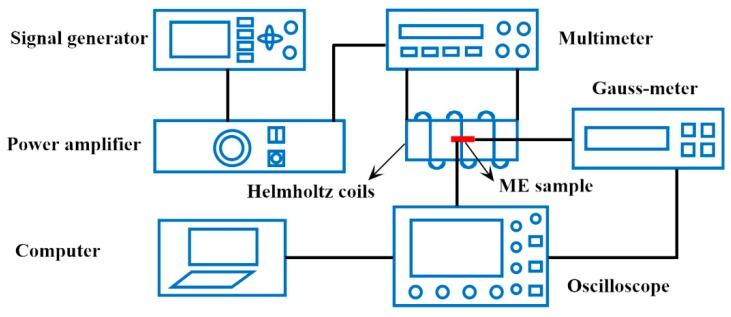
Schematic diagram of ME comprehensive testing system.

The experiment was carried out under normal temperature. The testing of the ME structure was completed on the ME comprehensive testing system, as shown in Figure 4. This test system was mainly composed of excitation of magnetic field signal and acquisition of output signal. The experimental setup is presented as follows. Firstly, place the sample in the center of the Helmholtz coils. Secondly, an AC signal is generated by a signal generator (ATF208, Atten, Shenzhen, Guangzhou, China) and inputted to a power amplifier (HFVA-62, Yingpu Magnetic Technology Development Co. Ltd., Changchun, Jilin, China). Then the input frequency is adjusted ([0, 200 kHz]), and the output voltages of the ME structures are measured with an oscilloscope (Agilent DSO9064A). Finally, the ME coefficient can be calculated by the definition $\alpha_{E}=\delta E/\delta H_{AC}=V_{P-P}/\left(2\sqrt{2}H_{AC}t_{P}\right)$, where *V_P−P_* is the peak-to-peak value of the output voltage, *H_AC_* is the virtual value of the AC magnetic field, and *t_p_* is the thickness of PZT layer [50].

## 3. Results and Discussion

### 3.1. The ME Coefficient in the A-Line Shape Terfenol-D/PZT Structure

**Figure 5:**
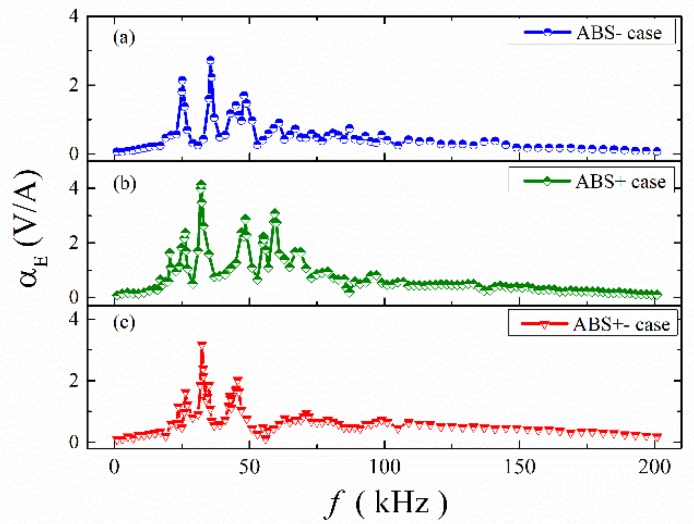
The frequency dependences of α_E_ for ME structures in different conditions: (**a**) ABS- case; (**b**) ABS+ case; (**c**) ABS+- case.

Firstly, the relation between the ME coefficient and frequency has been studied for the ME structures with ABS and mica trestles. Here, the alternating magnetic field was 79.6 A/m. The frequency changed from 0 to 200 kHz. Figure 5a–c shows the ME coefficient versus frequency for the ME structures with ABS trestles when the permanent magnet was put in different places. It is clear that a multi-peak phenomenon appeared in the ME structure. Table 1 shows the number of peaks and the resonance frequencies for the ME structure with and without NbFeB magnet. The data without NbFeB magnet comes from the study of Zhang et al. [34]. The DC magnetic field was 79.6 kA/m, which was provided by an electromagnet and a power supply (see Figure 3). For the ME structure with NbFeB magnet, multiple resonance peaks appeared in the frequency range 0–70 kHz. For frequencies over 70 kHz, there was basically no resonance phenomenon. For the ME structure without NbFeB magnet (*H_DC_* = 79.6 kA/m), resonance occurred when the frequency exceeded 70 kHz. Besides, resonance frequency also varied in three cases, as shown in Table 1. Moreover, the maximum ME coefficient was different in three cases. For example, the maximum ME coefficient in the ABS- case was 2.724 V/A at 35.6 kHz; for the ABS+ case, it was 4.142 V/A at 32.2 kHz; and for the ABS+- case, it was 3.173 V/A at 32.4 kHz.

**Figure 6:**
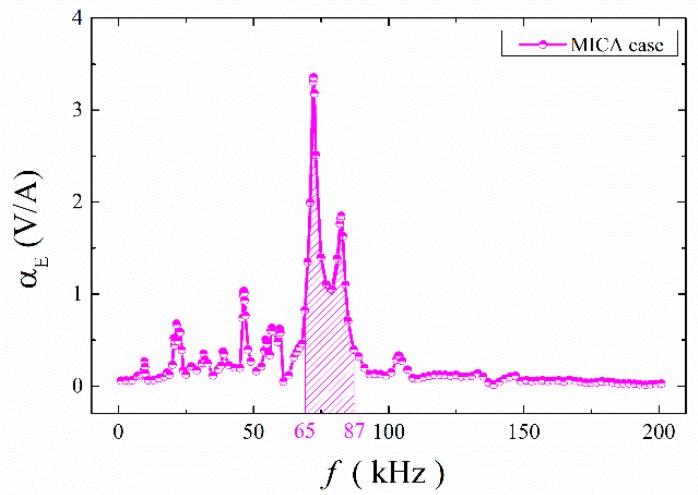
The frequency dependences of α_E_ for ME structures with mica trestles.

The ME coefficient versus frequency for the ME structures with mica trestle is shown in Figure 6. It can be seen that the multi-frequency phenomenon was also observed in these ME structures. Moreover, the ME coefficient maintained high values in the frequency domain 65~87 kHz, as shown by the shaded areas. It may be useful in designing a broadband ME energy harvester. The maximum ME coefficient is 3.355 V/A at 72.2 kHz. Table 2 shows the resonance frequencies for the ME structures with and without NbFeB magnet. The resonance frequencies for the ME structure without NbFeB magnet (*H_DC_* = 79.6 kA/m) were 21.0 kHz, 29.0 kHz, 39.0 kHz, 71.5 kHz, 83.0 kHz, and 105.0 kHz. For the ME structure with NbFeB magnet, in addition to resonance at the vicinity of the above six frequencies, resonance at 9.7 kHz, 46.5 kHz, 56.7 kHz, and 59.7 kHz was also generated.

### 3.2. Transient Signal

Next, we analyzed the structural frequency response characteristics at different applied frequencies. The applied frequency varied from 0 to 120 kHz for the two types of ME structures. In order to show the variation of voltage with time at low and high frequencies, we chose three frequencies in the frequency range 0–200 kHz. The considered frequencies were 0.8 kHz, 7 kHz, and 65 kHz, and the AC magnetic field was 79.6 A/m (1Oe).

**Figure 7:**
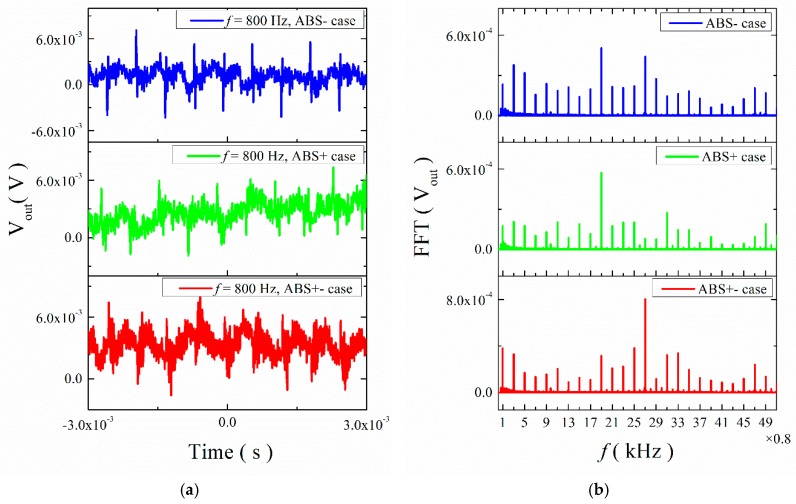
(**a**) Waveforms of ME voltage in the time domain for the ME structure with ABS trestles at 0.8 kHz; (**b**) the FFT spectral pattern for ME voltage.

**Figure 8:**
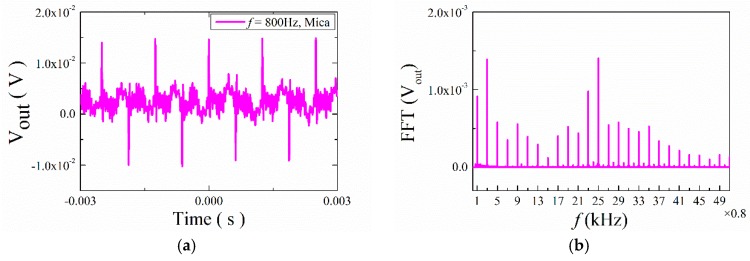
(**a**) Waveforms of ME voltage in time domain for the ME structure with mica trestles at 0.8 kHz; (**b**) the FFT spectral pattern for ME voltage.

Figure 7 shows the transient response curves of the output voltage V_out_ for the ME structures with ABS trestles under the low frequency. The input frequency was *f* = 0.8 kHz. No matter how the permanent magnet was placed on the ME structure, the transient curves were irregular curves. In order to study this condition further, the fast Fourier transform (FFT) was used to analyze the out voltage, as shown in Figure 7b. We observed that the frequencies of the output signals were 0.8 kHz, 2.4 kHz, 4 kHz, 5.6 kHz, etc. In other words, the frequencies of the output voltage were odd multiples of the input frequency. This means that the phenomenon of multiple frequencies appeared. Besides, it can be found that the frequency 15.2 kHz (19 times the input frequency) took up a great portion of the whole output frequencies in ABS- and ABS+ cases, and the frequency 21.6 kHz (27 times the input frequency) accounted for a higher percentage in the ABS+- case. Then we measured the output voltage for the ME structure with mica trestles and found the same phenomenon in this structure; the frequencies of 2.4 kHz (3 times the input frequency) and 20 kHz (25 times the input frequency) have large portions, as seen Figure 8.

These experimental phenomena show that the frequency of the structure appeared multiple response frequencies under low applied frequency. This was strongly related to the nonlinear properties of the component materials. Therefore, in the theoretical analysis, in the case of low frequency, the influence of the nonlinear characteristics of the material on the magnetoelectric effect must be considered.

**Figure 9:**
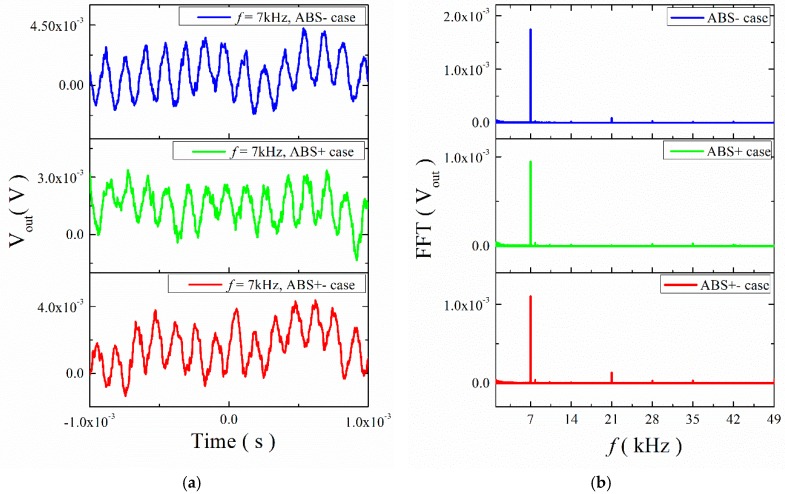
(**a**) Waveforms of ME voltage in the time domain for the ME structure with ABS trestle at 7 kHz; (**b**) the FFT spectral pattern for ME voltage.

**Figure 10:**
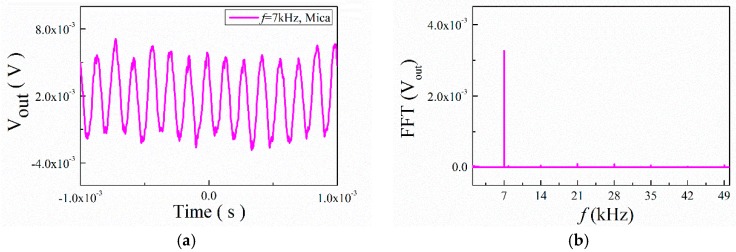
(**a**) Waveforms of ME voltage in the time domain for the ME structure with mica trestle at 7 kHz; (**b**) the FFT spectral pattern for ME voltage.

Next, we gradually increased the input frequency (*f* = 7 kHz) to study the transient response curves of the output voltage for the ME structures. The results are shown in Figure 9 and Figure 10. They show that the output signal with noise had a certain regularity and periodicity. A spectrum analysis of the curves shows that the output frequencies were 7 kHz, 14 kHz, 21 kHz, 28 kHz, etc. All output frequencies were multiples of 7 kHz. The signal of 7 kHz plays a primary role.

**Figure 11:**
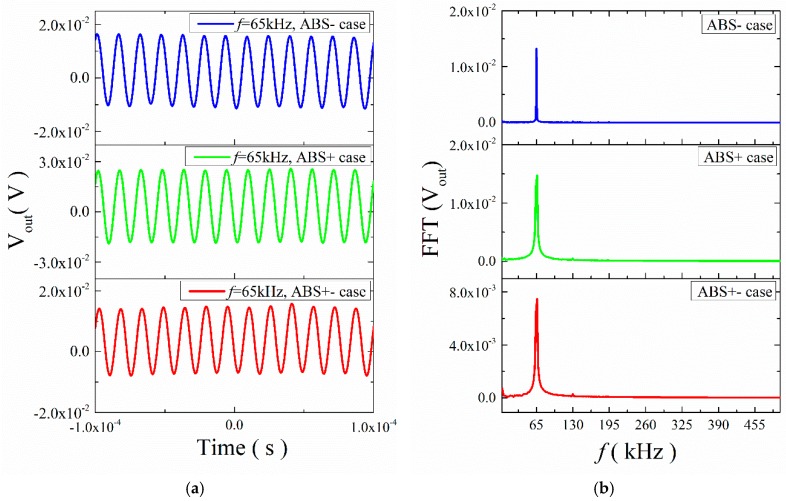
(**a**) Waveforms of ME voltage in the time domain for the ME structure with ABS trestle at 65 kHz; (**b**) the FFT spectral pattern for ME voltage.

**Figure 12:**
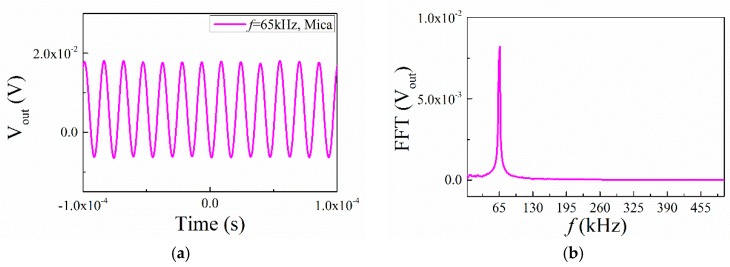
(**a**) Waveforms of ME voltage in the time domain for the ME structure with mica trestle at 65 kHz; (**b**) the FFT spectral pattern for ME voltage.

Then we investigated the transient ME response for the ME structures with mica trestles and ABS trestles under a higher frequency (*f* = 65 kHz). The results, shown in Figure 11 and Figure 12, indicate that the time domain waveforms changed in a sinusoidal fashion. The frequencies of the output signal were all 65 kHz, which is the same as the input frequency. It shows that under high frequency excitation, the response frequency of the structure was consistent with the applied frequency, and the frequency multiplication disappeared in the higher frequencies.

**Figure 13:**
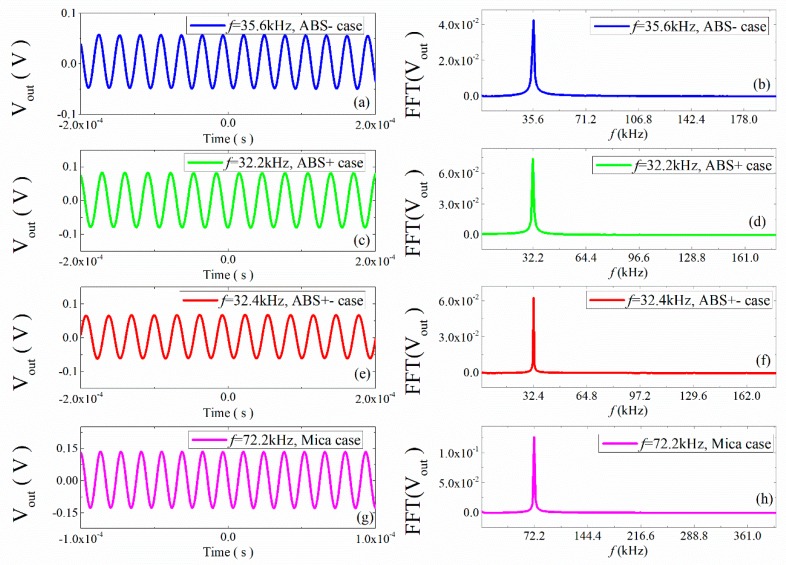
(**a**,**c**,**e**,**g**): Waveforms of ME voltage in the time domain for the ME structure at the higher resonance frequency; (**b**,**d**,**f**,**h**): the FFT spectral pattern for ME voltage.

To further illustrate the frequency characteristics of the output signal of the structure under resonance, we analyzed the frequency response characteristics of the four structures at the maximum magnetoelectric coefficient. At that point, the structure was in a resonance state. The output voltages of the four structures are shown in Figure 13a,c,e,g, and fast Fourier transforms of the voltage are shown in Figure 13b,d,f,h. It can be seen that although the structure was in a higher frequency environment, it was also in a resonance state, but the output frequency at this time was still the same as the input frequency; that is, at the resonance frequency, there were not multiple frequencies in the output signal of the structure. The phenomenon of multiple frequencies of the structure was indeed due to the nonlinearity of the material, but the applied excitation may also have caused the multiple frequency phenomenon.

### 3.3. The Effects of AC Magnetic Fields on ME Voltage

**Figure 14:**
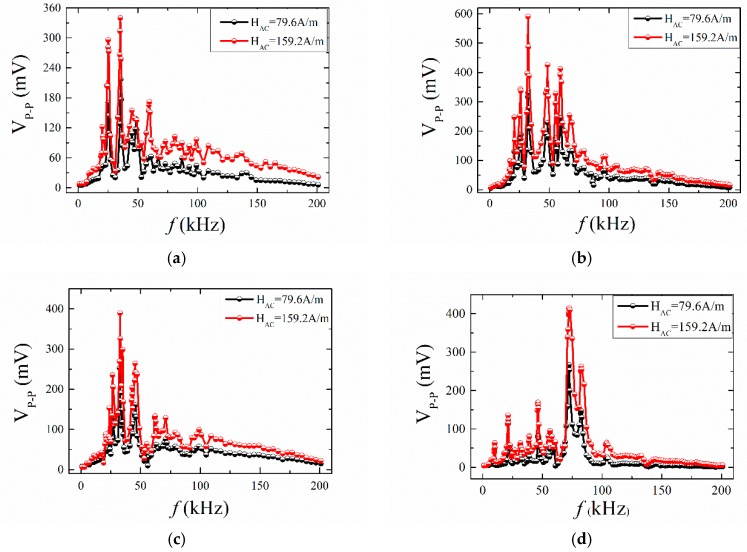
The frequency dependence of the self-biased ME voltage for ME structures at 79.6 A/m and 159.2 A/m; (**a**) ABS- case; (**b**) ABS+ case; (**c**) ABS+- case; (**d**) Mica case.

After that, we studied the influence of AC magnetic fields on the ME effect in the ME structures. In our experiments, the AC magnetic field was provided by a pair of Helmholtz coils. For the restrictions in the existing experimental conditions, the magnitude of the AC magnetic field could not reach a high value when the frequency was over 100 kHz. Here, the AC magnetic field was set to 79.6 A/m and 159.2 A/m, respectively. The frequency dependence of the ME voltage is shown in Figure 14. It was observed that the ME voltage at 159.2 A/m was higher than that at 79.6 A/m. The maximum of V_p-p_ researched 590 mV in the ABS+ case at 159.2 A/m. There were many resonance peaks in the frequency domain [0, 100 kHz]. The frequencies of the peak value of V_p-p_ were basically the same at 79.6 A/m and 159.2 A/m. For the ME structures with mica trestle, it can be seen that compared to the case at 79.6 A/m, the case at 159.2 A/m had a wider frequency domain.

### 3.4. The Finite Element Model of Vibration Mode and Magnetic Field Distribution of ME Structures

**Figure 15:**
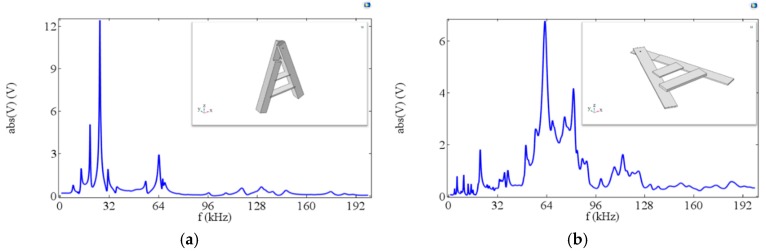
The relationship between the output voltage and frequency for A-line shape ME structures with (**a**) ABS trestles and with (**b**) mica trestles.

To explain the phenomena mentioned above, a finite element modeling analysis based on a commercial software COMSOL Multiphysics was performed for the A-line shape structures. Figure 15 shows the relationship between the output voltage and frequency for the A-line shape ME structure with ABS and mica trestles. The results show that both types of ME structures had multiple resonance frequencies. For the structure with ABS trestles, multiple common peaks appeared at 0–70 kHz. For the structure with mica trestles, the output voltage remained at high values in the 60–80 kHz range, which is consistent with the experimental results [34]. The existing theory shows that the effective material parameters (such as effective elastic modulus and effective piezomagnetic coefficient) of Terfenol-D materials change under the action of a magnetic field [51]. When a NdFeB permanent magnet is added to the structure, it may affect the performance of Terfenol-D due to a non-uniform magnetic field around it; hence, the addition of NdFeB may change the resonant frequency of the entire structure. This is one reason why the measured resonance frequency changed when the permanent magnets were placed at different positions in the experiment.

**Figure 16:**
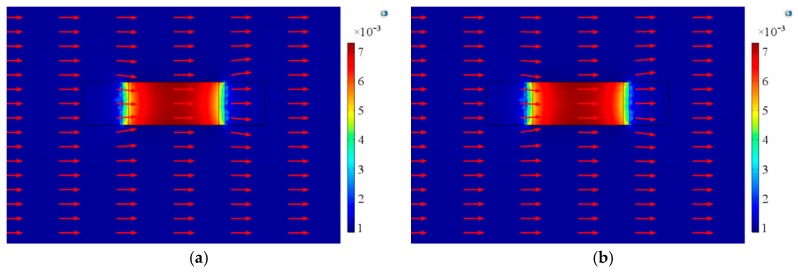
Magnetic flux distribution on (**a**) the upper and (**b**) lower surfaces of Terfenol-D under a uniform magnetic field.

Finally, we analyzed the magnetic flux distribution characteristics of the A-line shape ME structures under different magnetic fields. Figure 16 shows the magnetic flux distribution on the upper and lower surfaces of Terfenol-D material under a uniform DC magnetic field. There was no NdFeB material in the structure in this case. It can be seen that the magnetic field distribution of the upper and lower surfaces were the same. In the middle part of the Terfenol-D layer, the magnetic field was almost evenly distributed. Since the two sides of the material were fixed, the magnetic field distribution was uneven at the edges.

**Figure 17:**
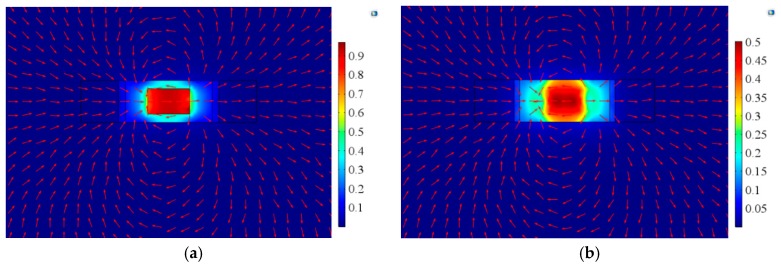
Magnetic flux distribution of Terfenol-D on (**a**) the surface near NdFeB and (**b**) the surface away from NdFeB under one NdFeB permanent magnet.

Figure 17 shows the magnetic flux distribution of the two surfaces of the Terfenol-D layer under one NdFeB permanent magnet, corresponding to the case of Figure 1a,b,d in the experiment. Figure 17a shows the magnetic flux distribution near one side of the permanent magnet, and Figure 17b shows the magnetic flux distribution away from the side of the permanent magnet. It can be seen that the magnetic field distribution around the permanent magnet was non-uniform. In the case of Terfenol-D, the magnetic field near the permanent magnet position was large, and the magnetic flux distribution in the entire Terfenol-D layer was uneven. In addition, since the two surfaces of Terfenol-D were different from the position of NdFeB, the magnetic flux distributions of the two surfaces were also different.

**Figure 18:**
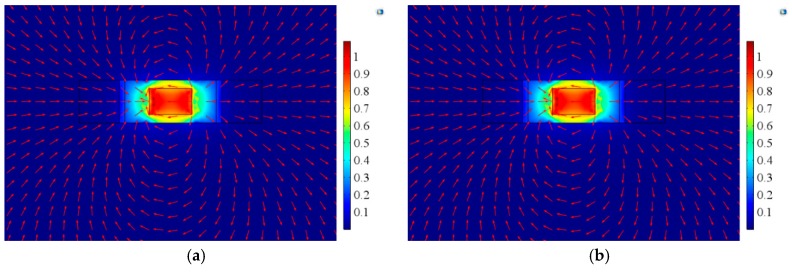
Magnetic flux distribution on (**a**) the upper and (**b**) lower surfaces of Terfenol-D under under two NdFeB permanent magnets.

Figure 18 shows the magnetic flux distribution of the two surfaces of the Terfenol-D layer under two NdFeB permanent magnets, corresponding to the case of Figure 1c in the experiment. Since the two magnets were symmetrical about the neutral layer of Terfenol-D, the magnetic flux distributions on the upper and lower surfaces of Terfenol-D were the same. Due to the uneven distribution of the magnetic field around NdFeB, the magnetic field distribution on any surface of Terfenol-D was also non-uniform. Overall, the magnetic field in the middle part was large, and the magnetic field at the edge position was small. By comparing Figure 17 to Figure 18, it is not difficult to find that the magnetic flux distribution on the same surface was different under the action of one and two permanent magnets.

Based on the above analysis, we can conclude that when the position of the permanent magnet is different, the magnetic field environment of the structure is different. The elongation of Terfenol-D is closely related to the applied magnetic field. Hence, when the permanent magnet is placed in different positions, it will affect the elongation of the entire Terfenol-D, which is the cause of the ME effect of the structures. As a consequence, the ME coefficient of the entire structure is different when the permanent magnet is placed in different positions.

## 4. Conclusions

In summary, the influence of NdFeB magnets on the ME effect in A-line shape Terfenol-D/PZT structures was investigated in this paper. The ME coefficient at different frequencies has been studied for the ME structures with ABS and mica trestles. The experimental results indicate that multiple resonance peaks exist in the A-line shape structures. The response frequencies are significantly affected by the position of the NdFeB permanent magnet in the ME structures. The maximum ME coefficient for ABS+ case was 4.142 V/A at 32.2 kHz, which is 60.3% of that at H_DC_ = 79.6 kA/m. For the ME structures with mica trestles, the ME coefficient maintained high values in the frequency domain 65~87 kHz. Fast Fourier analysis of voltage transient curves showed that the phenomenon of multiple frequencies appeared at a low frequency due to the nonlinear properties of the component materials. At the higher resonance frequency, there were no multiple frequencies in the output signal of the structure. This indicates that the frequency characteristics of the output voltage not only depend on the nonlinear characteristics of the component materials but also on the external excitation. Moreover, the influence of AC magnetic fields on the ME voltage was studied for the two structures. The amplitude of the AC magnetic field only changed the magnitude of the voltage without changing the resonant frequencies. Finally, a finite element modeling analysis was performed to analyze and explain the phenomena in the experiment. The simulation results show that the magnetic field distribution on the surface of Terfenol-D was non-uniform due to the uneven distribution of the magnetic field around NdFeB. When the position of the permanent magnet was different, the magnetic field environment of the structure was different, and this in turn led to different ME coefficients in the A-line ME structures. With this in mind, one can tune the resonant frequency of ME system by changing the location of the external permanent magnet, and thus maximize the ME performance of the ME structure. This may provide a useful basis for the improvement of ME coefficients and for the optimal design of ME sensors/harvesters.

## Figures and Tables

**Table 1:** The resonance frequencies in ME structures with ABS trestles.

ME Structure	ABS- Case	ABS+ Case	ABS+- Case	Without NbFeB (H_DC_ = 79.6 kA/m) [34]
Response frequencies (kHz)	25.2, 35.6, 45.0, 48.0, 61.0	20.6, 26.2, 32.2, 48.5, 55.2, 59.4, 67.0	23.7, 26.4, 32.4, 35.0, 42.8, 45.6	21.0, 37.6, 46.1, 57.3, 81.2, 87.0
*α_E,max_* (V/A)	2.724	4.142	3.173	6.877

**Table 2:** The resonance frequencies in ME structures with mica trestles.

ME Structure	With NbFeB	Without NbFeB (*H_DC_* = 79.6 kA/m) [34]
Response frequencies (kHz)	9.7, 21.6, 31.5, 38.7, 46.5, 56.7, 59.7, 72.2, 82.5, 104.0	21.0, 29.0, 39.0, 71.5, 83.0, 105.0
Broadband range (kHz)	65–87	65–87
